# Poorer Intermittent Sprints Performance in Ramadan-Fasted Muslim Footballers despite Controlling for Pre-Exercise Dietary Intake, Sleep and Training Load

**DOI:** 10.3390/sports5010004

**Published:** 2017-01-06

**Authors:** Abdul Rashid Aziz, Ahmad Munir Che Muhamad, Siti Raifana Roslan, Nazirah Ghulam Mohamed, Rabindarjeet Singh, Michael Yong Hwa Chia

**Affiliations:** 1 Sport Science Centre, Singapore Sports Institute, Sport Singapore, Singapore 397630, Singapore; 2 Physical Education and Sport Science, National Institute of Education, Nanyang Technological University, Singapore 637616, Singapore; michael.chia@nie.edu.sg; 3 Advance Medical and Dental Institute, Universiti Sains Malaysia, Penang 13200, Malaysia; ahmadmunir@usm.my (A.M.C.M.); raifanaroslan@gmail.com (S.R.R.); nazirah_gm@moh.gov.my (N.G.M.); r.singh1955@gmail.com (R.S.); 4 Faculty of Medicine, Asian Institute of Medicine, Science and Technology University, Kedah 08100, Malaysia

**Keywords:** intermittent fasting, fatigue, dehydration, placebo, football, intermittent sports

## Abstract

This study examines the effects of Ramadan fasting on sprint performance during prolonged intermittent exercise in trained Muslim footballers, under controlled pre-exercise conditions. A within-group, cross-over study design with two non-fasted or Control trials performed before (i.e., CON1) and after (CON2) the Ramadan month, and with the Ramadan-fasted (RAM) trials performed within the Ramadan month. After familiarization, 14 players completed a modified 60-min (4 × 15-min exercise blocks interspersed with 3-min intervals) of the Loughborough Intermittent Shuttle Test (mLIST) of fixed speeds of walking, jogging, running, but with all-out effort sprints. During the interval periods, capillary blood glucose and blood lactate measures were taken, rectal and skin temperatures were recorded and maximal voluntary isometric contractions (MVIC) of the dominant leg and hand-grip were performed to provide some indication to the cause(s) of ‘fatigue’ during exercise. Players were provided with standardized 24-h pre-packed meals prior to all trials. Sleep hours were objectively assessed and perceived training loads were monitored and these were equivalent between RAM and CON trials. Sprint times throughout mLIST were significantly faster in both CON1 and CON2 as compared to RAM trials (all *P* < 0.017; *d* = small to moderate), and this poorer performance in RAM was observed as early as during the first 15-min of the mLIST. Blood markers, MVIC and thermoregulatory results were not substantially different between both CON and RAM trials. In conclusion, despite similarities in dietary intake, sleeping hours and training loads between conditions, results still indicate that Ramadan fasting had an adverse effect on prolonged intermittent performance. Nocebo effects plays a dominant role during exercise in the Ramadan-fasted state.

## 1. Introduction

The recent reviews on the effects of Ramadan fasting and exercise performance indicated mixed findings [1,2], with some studies indicating an adverse impact and while some investigations indicating no differences in the exercise performances performed in Ramadan-fasted vs. performed in the non-fasted state. A potential reason for the diverse findings is that in many of these studies there was the lack of control for variables such as the subjects’ pre-exercise dietary intake, sleep hours, and training load. The timings and caloric content of the *sahur* meal (i.e., food and fluid taken just before the commencing of the day’s fast) undertaken in these studies could have an impact on metabolism during muscular contractions [3]. For example, reduced levels of muscle glycogen as a result of substantially reduced total calories intake are shown to result in lower rates of glycogenolysis during subsequent sustained exercise [4,5]; or in the worst case, if the *sahur* meal during Ramadan was even not consumed in the study [6]. Alteration of sleeping hours over days during the Ramadan month can drastically shift the bodily circadian rhythm, leading to smaller amplitude of fasted individual’s body temperature [7,8]. Such a shift in circadian rhythm can further influence heart rate during exercise and hormones secretion and can therefore indirectly impact the strength of muscular contractions [8,9,10]. The reduced frequency and intensity of training commonly reported in Ramadan month can also lead to detraining effects during exercise, occurring late in Ramadan [11,12]. Therefore, there is clearly a need to control or account for these three variables of pre-exercise food and fluid intake, sleep and training stimulus when examining the impact of Ramadan fasting on exercise performance.

The aim of the present study was to examine the impact of Ramadan fasting on sprints performance during prolonged intermittent exercise under ecological-valid but controlled settings where confounding factors such as endogenous fuel, lack of sleep and training load are equivalent to Control conditions. A secondary aim of the study was to determine the possible reasons for the potentially adverse effects of Ramadan fasting on exercise performance, if observed. In this regard, maximal voluntary isometric contraction (MVIC) of single-knee extension (i.e., exercised muscle) and MVIC of hand-grip (i.e., non-exercised muscle) were conducted during instituted rest-breaks throughout exercise. Players’ rectal and skin temperatures were also monitored to compare individuals’ thermoregulatory responses during exercise in the Ramadan-fasted and non-fasted state. It was hypothesised that Ramadan fasting would have a negative impact on the Muslim players’ sprint performance, maximal strength and thermoregulatory responses during prolonged intermittent exercise.

## 2. Methods

### 2.1. Participants

Sixteen male trained Muslim football players were recruited for the study. Two players however did not complete all procedures, and thus only 14 players’ [mean ± SD; age 21.8 ± 2.4 year; stature 170 ± 5 cm; body mass 67.1 ± 11.0 kg; estimated maximal aerobic power (${\dot{V}O}_{2\;\text{max}}$) 50.1 ± 4.5 mL·kg^−1^·min^−1^; maximum heart rate (HR_max_) 188 ± 10 b·min^−1^; and competitive experience 5.0 ± 1.4 year] data were analysed. All players are of the Muslim faith and have had performed the annual religious Ramadan fast for the past 10 to 12 years. Participants were athletes from a local university football team and the squad was in mid-competition playing in the second tier of the State League. The squad was training between 3–5 times a week, between 60 and 90 min per session, and played a competitive match at the end of the week. Training sessions were typically scheduled either in the late afternoon or early evening period and consisted of sport-specific technical drills and small-sided games. All players were local-born and are acclimatised to exercising in the hot and humid environment. All players provided written informed consent before participating. The study was approved by the Nanyang Technological University Ethics Committee (IRB 12-04/02) and was conducted in accordance with the Declaration of Helsinki.

### 2.2. Experimental Design

**Figure 1:**
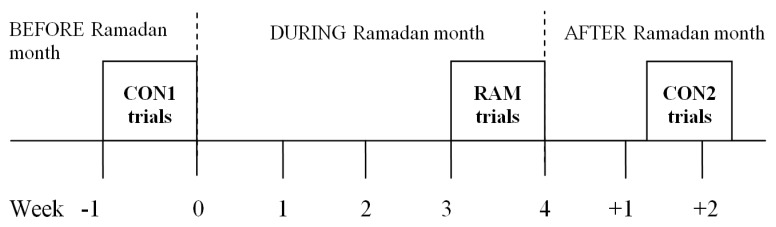
The present study’s time line. Non-fasted or control (CON1 = before Ramadan month, CON2 = after Ramadan month) and Ramadan-fasted (RAM = in the Ramadan month) exercise trials (*N* = 14).

A within-subject repeated-measures experimental design where each participant served as his own control was used. Players performed a 60-min modified Loughborough Intermittent Shuttle Test (mLIST) on four separate occasions: the first as familiarization, the second before the Ramadan month (i.e., first Control trial or CON1), the third occasion during Ramadan (RAM trial), and the final session was after the Ramadan month (second Control trial or CON2). CON1 trials were conducted during the last week before the Ramadan month and the CON2 trials were performed between 10 to 15 days after the end of the Ramadan month. The RAM trials were conducted within the last week of the Ramadan month (Figure 1). Comparisons were made between performances and physiological responses of players in CON1 and CON2 trials with that of RAM trial. The squad’s coach was briefed to ensure that the tested players’ training session 24 h prior to their experimental trial was of low intensity to ensure players did not suffer from accumulated fatigue. The study was undertaken to coincide with the actual month of Ramadan in which the duration of daily fasting was from ~05:45 to ~19:45 (a total of ~14 h). All exercise trials commenced at ~18:00 (for the RAM trial, players would have fasted for at least ~12 h). The trials were conducted in an enclosed gymnasium (but with high roof and walls but non air-conditioned) with sprung wooden flooring. Environmental conditions during all exercise trials were taken at 15-min intervals using a portable weather station (Kestrel 4000 Nelson Kellerman, Boothwyn, PA, USA). The ambient temperature and humidity for the three experimental trials ranged from 28–33 °C and 62%–82%, respectively.

### 2.3. Familiarisation

Players completed two familiarisation sessions during which all the experimental procedures and questionnaires were explained to them. Players then completed a multi-stage shuttle run (or Beep) test to determine their estimated ${\dot{V}O}_{2\;\text{max}}$ and HR_max_. The average ${\dot{V}O}_{2\;\text{max}}$ of the group was then used to calculate the estimated speeds at which players will perform the locomotor activities during the mLIST. In the second familiarization session, player completed two 15-min blocks of the mLIST and practised the maximal muscular exertions tests.

### 2.4. Modified Loughborough Intermittent Shuttle Test (mLIST)

**Figure 2:**
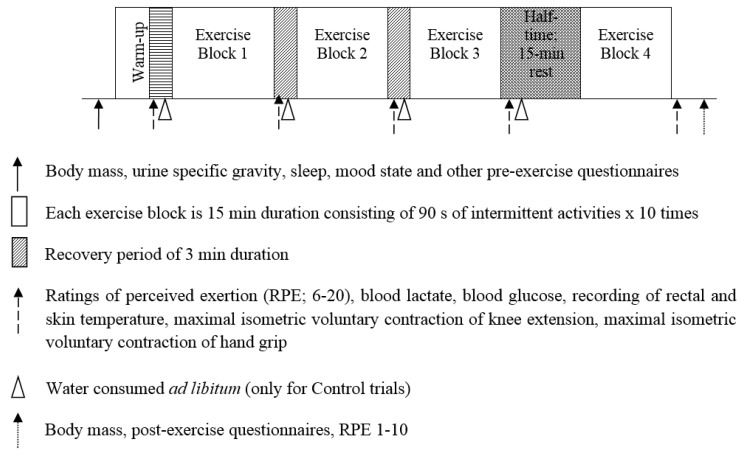
Schematic representations of the exercise trial and measurements time-points.

In the original LIST, the participant run between two lines, 20 m apart, at varying locomotor speeds for 75 min (5 × 15-min exercise blocks) with 3 min recovery between blocks, followed by a run to exhaustion, for a total duration of between 85–100 min [13]. For the present study, a modification of the LIST (i.e., mLIST) was used. Firstly, the same speeds of locomotor activities were imposed across all players. Secondly, the duration of the mLIST was shortened to 60-min (4 × 15-min exercise blocks) with the run to exhaustion component omitted. The mLIST protocol was deliberately shortened to minimise the influence of endogenous muscle glycogen on exercise performance in the study, as it has been shown that at the end of a 90-min football match, there was a substantial reduction in endogenous muscle glycogen stores [14]. Several physical and physiological measures were taken at the start, during the 3-min breaks between the four exercise blocks and at the end of the 60-min exercise (Figure 2).

**Figure 3:**
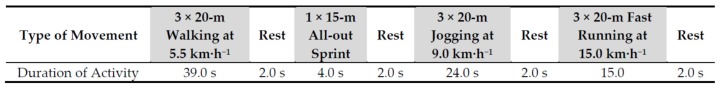
The schematic representation of the one 90 s cycle of intermittent shuttle movement patterns covering a distance of 195 m in the modified Loughborough Intermittent Shuttle Test (mLIST). The above cycle was completed 10 times to form one exercise block of 15 min in duration.

One cycle of intermittent shuttle movement patterns of the mLIST was as follows (Figure 3): walking 3 × 20-m shuttle at 5.5 km·h^−1^, followed by a 2.0 s passive recovery to allow the player time to move into the “ready position” for the next activity, followed by 1 × 15 m all-out maximal sprint effort followed by a 2.0 s passive recovery, then jogging 3 × 20-m shuttle at 9.0 km·h^−1^ followed by a 2.0 s passive recovery, and fast running 3 × 20-m shuttle at 15.0 km·h^−1^, followed by a 2.0 s passive recovery; and continue with the re-start of the walking pattern. Each cycle of intermittent shuttle movement patterns of activities lasted 90 and the cycle was repeated 10 times continuously to form a single 15-min exercise block, which was followed by a 3 min break interval. Players performed four exercise blocks of 15 min each (i.e., a total duration of 60 min) with a half-time break of 15 min at the end of the block 3 to emulate the duration pattern of a football match. Players followed the pattern of movements, i.e., walk, jog, or fast run dictated by audible beeps which have been pre-recorded onto a compact disc, except for the sprinting component where the player sprinted the 15-m distance as fast as possible, i.e., all-out maximal effort. The total distance covered for each 15-min exercise block is 1950 m and thus over the four 15-min exercise blocks of the mLIST, the player covered a total distance of 7800 m. If the mLIST exercise protocol is extended to 90 min, the total distance covered would have been 11,700 m, which is within the expected range of a full football match of 10–12 km [15]. Furthermore, in the mLIST, 3000 m (or ~38%) of the total distance covered was in the high-intensity zone (i.e., fast running and all-out sprint); which suggests that the mLIST was of greater intensity relative to an average match play.

### 2.5. Sprint Performance during mLIST

In the mLIST, sprints were initiated from a standing position, 20 cm behind the timing start gate. Sprint times were recorded with timing-gates (Speed Light Sports Timing system; Swift Performance Equipment, Lismore, NSW, Australia) to an accuracy of 1/100th of a second. The mean sprint time for each 15-min block of exercise, i.e., averaged over the 10 sprints, was used as the criterion measure of the player’s prolonged intermittent exercise performance. The coefficient of variation (CV) for the sprints performance in the original LIST was found to be <2% [13]. The use of sprint times as the criterion measure is supported by previous studies indicating the quality of the player’s sprints as a performance variable that differentiates the elite from well-trained players [16,17], and is deemed a valid measure of a player’s physical performance in the modern game of football [14,15]. In addition, although sprinting constitutes only ∼1% to ∼12% of the distance covered during match play, players’ sprinting ability may define outcomes of decisive situations of football matches [18].

### 2.6. Assessment of Muscular Exertion Capability in Exercised and Non-Exercised Muscles

Two maximal muscular measures were performed at pre-exercise (after the completion of the warm-up), and at the end of each block of exercise in the mLIST (Figure 2). These tests were the player’s maximal voluntary isometric contraction (MVIC) of the single leg (dominant) knee extension (i.e., lower limb and deemed as the exercised muscle) and hand-grip (upper limb and deemed as the non-exercised muscle). The MVIC assessment of the knee extensor used a load cell force-transducer (DBBP 200, Bongshin Loadcell Co. Ltd., Seoul, Korea) with the players in a secured seated position. One end of the load cell was attached to a non-elastic ankle strap situated just ~3 cm proximal to the medial and lateral malleoli of the dominant leg ankle with the other end of the load cell by a metal cable-wire, which was attached to an immovable secured point in the bar-stand of a purposely-built chair. During the test, the player was seated upright with the knee joint angle of ~100° on the edge of the table. Player’s body remained upright and arms folded across the chest throughout the data collection. On command, player extended his lower leg as fast and powerful as possible and to maintain this maximal exertion for a ~4 s duration while receiving verbal encouragement throughout. The highest force displayed on an amplified digital indicator (Rinstrum Pty. Ltd., Model 2100EX, Brisbane, Australia) in Newtons (N) was taken as the player’s MVIC score of the knee extensor muscle. The MVIC of the dominant hand was measured with a hand-grip dynamometer (T.K.K. 5101 Takei, Tokyo, Japan) with standardized protocols. The dynamometer handle was adjusted for each individual so that it fitted onto the hand with the top of the handle resting on the middle phalange while the base rested on the proximal end of the metacarpals. From a position with the hand held above the head, elbow extended, the arm moved through an arc until it rested by the side of the body, all the while squeezing the dynamometer maximally. The highest value was taken as the hand-grip MVIC (in kilogram force, kgf). For all trials, players were instructed to give their maximal effort and no visual feedback of result was provided during the tests. Due to the limited time of only 3-min between exercise block 1 and block 2 and between block 2 and block 3, only a single trial of the knee extension and hand-grip measure (with 30-s rest between trials) was conducted. For pre-exercise, and after exercise block 3 and block 4 where there was ample time available, two trials (with 60-s passive rest between trials) of each of the two muscular tests were completed. For all trials, the best score attained at each interval time-point was retained for analysis.

### 2.7. Rectal (T_re_) and Skin Temperature (T_sk_)

Thermoregulatory measures were taken at post warm-up and at the end of every exercise block. *T*_re_ was measured via a disposable rectal thermistor probe (YSI 400, Mallinckrodt Medical, Kansas City, MO, USA) inserted 10 cm past the anal sphincter. The output of the temperature probe was sampled with a digital thermometer (Philips Xm 8530/10, Cole Parmer, Vernon Hills, IL, USA). *T*_sk_ was measured at four different sites: the midpoint of the right pectoralis major (chest), midpoint of the triceps brachii lateral head (tricep arm), right rectus femoris (thigh) and right gastrocnemius lateral head (calf); using the iButton^TM^ temperature sensors (Maxim Integrated Products, Sunnyvale, CA, USA) [19]. The sensors were secured in position with special tape (Opsite Flexifix, Smith and Nephew, London, UK) which allows for the evaporation of sweat. *T*_re_ was used as the representation of body core temperature and mean *T*_sk_ was calculated using a weighted average of four sites [20]: chest 30%, triceps 30%, thigh 20%, and calf 20%.

### 2.8. Pre-Exercise Measures

Upon arrival to test location, the player voided his bladder and provided a urine sample for the measurement of urine specific gravity (Usg; PAL-10S, Atago Co. Ltd., Tokyo, Japan) to determine the player’s pre-exercise hydration status. Thereafter, several other measures such as body mass, blood lactate, blood glucose, and their sleep, diet and training load questionnaires were conducted. Capillary blood samples were taken via the finger prick method to determine resting pre-exercise blood glucose (Accu-chek Performa, Roche Diagnostics GmbH, Mannheim, Germany) and blood lactate concentrations (Lactate Pro, Arkray Inc., Kyoto, Japan). The same procedures were used to measure the same blood markers at the end of each block of exercise. Each player was then equipped with a HR strap, rectal probe, and skin thermistors. Players then performed a standardized warm-up consisting of 8-min of self-paced jog, 5-min of dynamic stretching focusing on the lower limbs, followed by two cycles of the mLIST. Player then rested for 2-min before performing the baseline measures of the muscular exertion tests. The player then rested passively for 5-min before commencing the mLIST exercise.

### 2.9. Fluid Intake during Exercise

The player’s body mass (in shorts only and toweled dry) was also taken before and after the 60-min exercise on a weighing scale with an accuracy of ±50 g (Seca 874, Gmbh and Co., Hamburg, Germany). Players were allowed to consume water *ad libitum* (only for CON1 and CON2 but not for RAM trials) from pre-prepared water bottles during the interval periods. The bottles were weighed with an electronic balance (KD-160, Tanita, Tokyo, Japan) at end of the exercise to calculate the player’s fluid intake. The player’s sweat loss during exercise was then determined, taking into account of body mass change and fluid consumed during exercise (for CON trials only), with the formula: sweat loss = (pre-body mass − post-body mass (kg) + fluid ingested (mL) − urine loss (mL).

### 2.10. Heart Rate (HR) and Ratings of Perceived Exertion (RPE)

Players rated their subjective RPE using Borg’s 6–20 scale [21] at pre-exercise and at the end of each exercise block. In addition, the Borg’s categorical scale of 1–10, as a measure of the overall psycho-physical exertion over the entire 60-min exercise trial, i.e., session-RPE (s-RPE) was administered at post-20 min of the mLIST [21]. Heart rate via short-range telemetry (Polar Electro Oy, Kempele, Finland) was continuously recorded during trial.

### 2.11. Profile of Mood State and Perceived Readiness Questionnaires

At pre-exercise, the players completed the validated Brunel Mood State (BRUMS) profile [22]. Players also rated their perceived level of subjective feeling for their readiness to exercise, tiredness, alertness, and ability to concentrate using a visual analogue scale, as was similarly done in previous Ramadan fasting and exercise investigation [23].

### 2.12. Dietary Intake

All participants were provided with pre-packed cooked meals (AMDI Catering Pte Ltd., Penang, Malaysia) for the 24-h period prior to all their exercise sessions. The meal plan provided an estimated energy of ~11 MJ consisting of 6.0 g·kg body mass^−1^ of carbohydrate, 3.0 g·kg body mass^−1^ of protein, and 3.5 g·kg body mass^−1^ of fats per day, and about 3.5 L of fluids consisting of bottled mineral water, commercial sweetened beverages, milk and fruit juice. The players were then provided with specific instructions by a certified nutritionist on the distribution of the specific amount of foods and fluids to be consumed over three meals throughout the 24 h period prior to the three exercise trials (Table 1). Therefore, although meals and fluids were taken at different times of the day between the CON and RAM periods, the amount consumed in the 24 h prior to each experimental trial was the same within each player and relatively equivalent across participants.

### 2.13. Sleep

In many previous Ramadan exercise studies, participants verbally reported their bedtime, but it was not unclear of the exact time that they actually fell asleep. To ensure an objective and reliable measure of sleep quantity in the present study, each player wore an actigraph watch (Actiwatch 2, Philips Respironics Inc., Bend, OR, USA) for 24 h prior to his exercise trial. The actiwatch has been used to quantify the sleep and wake behaviour of athletes [24] where the actiwatch sleep software was used to determine the wearer’s “total sleep time”, which was the same criteria of sleep duration used in the present study. Actigraph has been shown to correlate well with the gold standard of sleep assessment, i.e., polysomnography; with intraclass correlation coefficient of 0.76 for total sleep time [25]. In addition, players were also queried on their level of daytime sleepiness using the Karolinska Sleepiness Scale (KSS) [26], which has been previously used in Ramadan exercise study [23].

### 2.14. Training Load

To ensure that training stimulus would not compound the present study’s findings, the squad’s coach agreed to maintain a consistent training load across the Ramadan month. The coach planned and conducted these sessions. The players were also required to log their attendance and complete their s-RPE (rating 1–10) [19] at the end of the sessions in a diary for one week prior to their CON1, RAM and CON2 trials. The data was used to determine the players’ weekly training load [27].

## 3. Statistical Analyses

The Statistical Programme for Social Sciences 15.0 for Windows (SPSS Inc., Chicago, IL, USA) was used for all statistical analyses. All data were reported as mean ± SD. The level of statistical significance for primary effects was set at *P* < 0.05. The assumption of sphericity was checked by Mauchly’s test of sphericity and whereby violations occurred, the Greenhouse-Geisser Epsilon was applied. Differences in measured variables between CON1, CON2 and RAM trials were determined using one-way analysis of variance (ANOVA). Data with continuous time component were determined using ANOVA test with two-way repeated measures (Trial: CON1, CON2 and RAM × Time: exercise block 1 to block 4). If a significant main effect was found, post-hoc paired *t*-test was used to detect where the differences occurred with a Bonferroni correction for multiple comparisons, *P* = 0.05/3 = 0.017. In addition, effect size (*d*) is utilised to determine the practical meaningfulness of the differences (Cohen’s *d*) for some of the variables. The magnitude of *d* was classified as trivial (where <0.2), small (>0.2–0.6), moderate (>0.6–1.2), large (>1.2–2.0) and very large (>2.0–4.0) based on guidelines promoted by Batterham and Hopkins [28].

## 4. Results

### 4.1. Sprint Times in the mLIST

**Figure 4:**
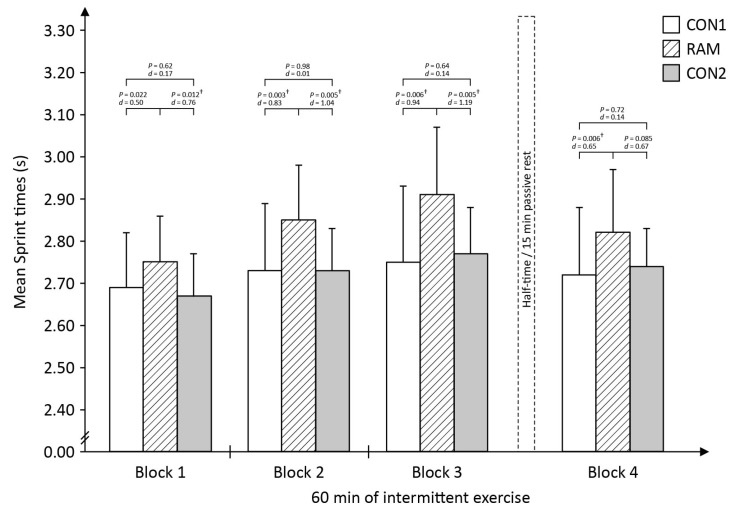
Players’ mean 15-m sprint times during the modified Loughborough Intermittent Shuttle Test (mLIST) exercise in the non-fasted or control (CON1 = before Ramadan month, CON2 = after Ramadan month) and Ramadan-fasted (RAM = in the Ramadan month) exercise trials (*N* = 14). † Post-hoc significant differences, *P* < 0.017.

Figure 4 shows the mean sprint times in each exercise block over the three experimental trials. The players’ overall sprint performance significantly declined with duration of exercise during all three experimental trials (*F*_3,39_ = 29.57, *P* < 0.001). More specifically, mean sprinting times during exercise block 3 and block 4 were significantly slower than in block 1. Sprinting times during block 4 (after 15-min passive rest) was similar to block 1. There was no significant interaction effect for mean sprint times (*F*_6,78_ = 1.85, *P* = 0.10). More importantly, there was a primary effect of mean sprint times for trial (*F*_2,26_ = 7.51, *P* = 0.003). Post-hoc analysis indicated that mean sprint times were generally slower in RAM relative to CON1 and CON2 trials throughout the four blocks of exercise, in particular during block 2 and block 3 (all *P* < 0.017; Figure 4). The *d* differences in mean sprint times between RAM and CON trials ranged from small to moderate (from 0.50–1.19; Figure 4). Post-hoc analysis indicated no significant differences in the mean sprint times between CON1 and CON2 trials across all four blocks of exercise (all *P* > 0.017).

### 4.2. Maximal Voluntary Isometric Contraction (MVIC) Tests

**Figure 5:**
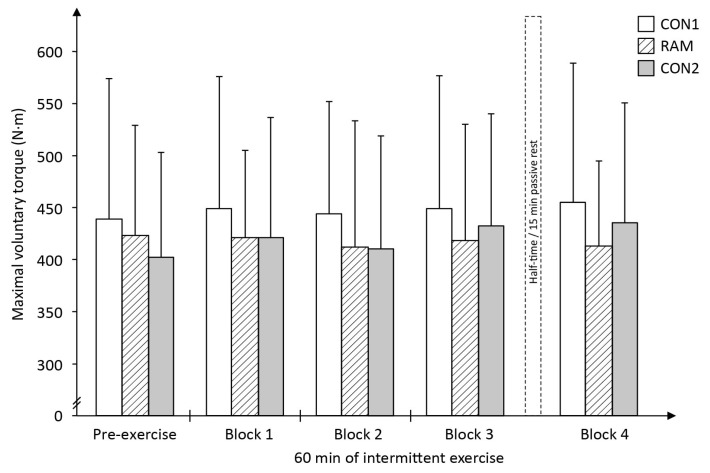
Players’ maximal voluntary isometric contraction (MVIC) of the dominant leg knee extension (i.e., exercised muscles) during the modified Loughborough Intermittent Shuttle Test (mLIST) exercise in the non-fasted or control (CON1 = before Ramadan month, CON2 = after Ramadan month) and Ramadan-fasted (RAM = in the Ramadan month) exercise trials (*N* = 14).

**Figure 6:**
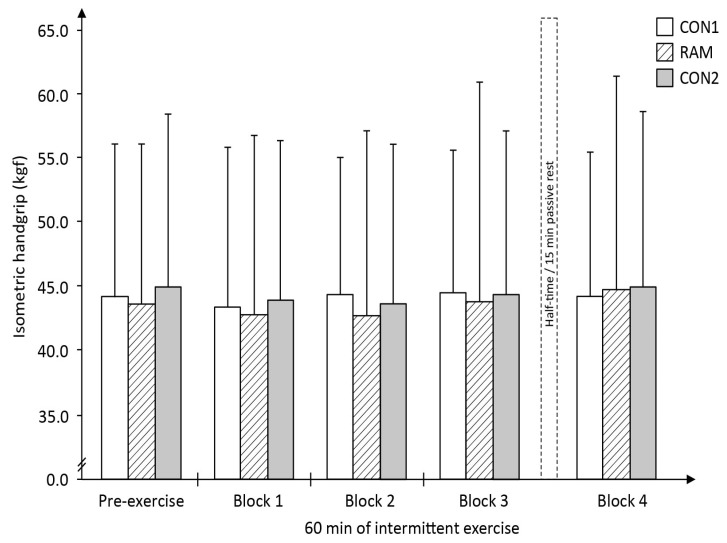
Players’ maximal voluntary isometric contraction (MVIC) of the dominant hand-grip (i.e., non-exercised muscles) during the modified Loughborough Intermittent Shuttle Test (mLIST) exercise in the non-fasted or control (CON1 = before Ramadan month, CON2 = after Ramadan month) and Ramadan-fasted (RAM = in the Ramadan month) exercise trials (*N* = 14).

It was expected that there would be a decline in the MVIC values in the lower limb due to fatigue from the physical exertion performing the mLIST, but no changes to the MVIC of the upper limb, which was exposed to minimal levels of physical exertion. The results show that there were no significant differences in the knee extension MVIC either for trial (*F*_2,26_ = 1.64, *P* = 0.214), or time (*F*_4,52_ = 1.21, *P* = 0.318), or interactions (*F*_8,104_ = 0.86, *P* = 0.55; Figure 5). Similarly, there were no significant differences in hand-grip MVIC for either trial (*F*_2,26_ = 1.22, *P* = 0.31), or time (*F*_4,52_ = 2.32, *P* = 0.069), or interactions (*F*_8,104_ = 0.56, *P* = 0.81; Figure 6).

### 4.3. Rectal and Skin Temperatures

Complete thermoregulatory measures were obtained only in 11 players due to dislodged rectal probes and/or skin thermistors during exercise. Both *T*_re_ and *T*_sk_ in all three experimental trials increased throughout mLIST (*F*_2.1,21.8_ = 233.49, *P* < 0.001; Table 2). However, there were no significant differences in *T*_re_ for trial (*F*_2,20_ = 0.99, *P* = 0.39) and interaction (*F*_3.6,35.7_ = 1.85, *P* = 0.15). Similarly, for *T*_sk_, there were no significant primary effect of trial (*F*_2,20_ = 2.52, *P* = 0.11), time (*F*_1.5,15.4_ = 0.09, *P* = 0.23) and interaction (*F*_2.5,25.4_ = 0.46, *P* = 0.08).

### 4.4. Heart Rate

**Figure 7:**
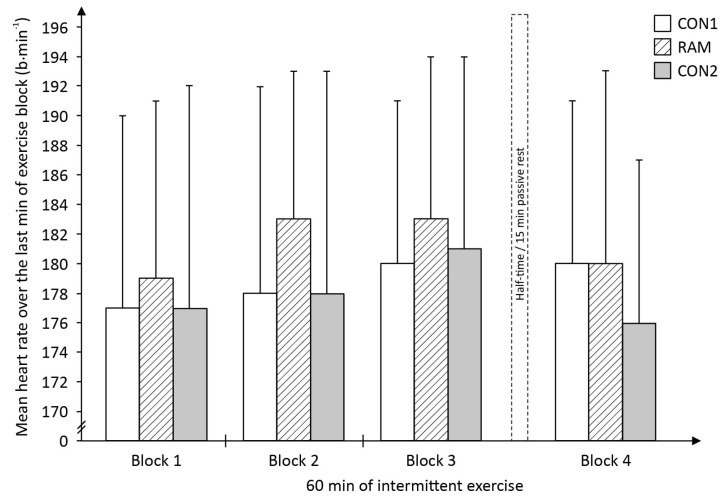
Players’ mean heart rate (HR) for the last min of the exercise block during the modified Loughborough Intermittent Shuttle Test (mLIST) in the in the non-fasted or control (CON1 = before Ramadan month, CON2 = after Ramadan month) and Ramadan-fasted (RAM = in the Ramadan month) exercise trials (*N* = 14).

The mean HR during the last minute of each exercise block in all three experimental trials tended to progressively increased from exercise block 1 to block 3 (Figure 7). Mean HR during block 4 tended to revert to levels in block 1. A two-way ANOVA indicated no statistical significant primary effects for either trial (*F*_2,26_ = 0.71, *P* = 0.49), or time (*F*_3,39_ = 2.04, *P* = 0.13). However, there were significant interactions effect (*F*_6,78_ = 7.51, *P* = 0.003).

### 4.5. Ratings of Perceived Exertion (RPE) and Session-RPE

**Figure 8:**
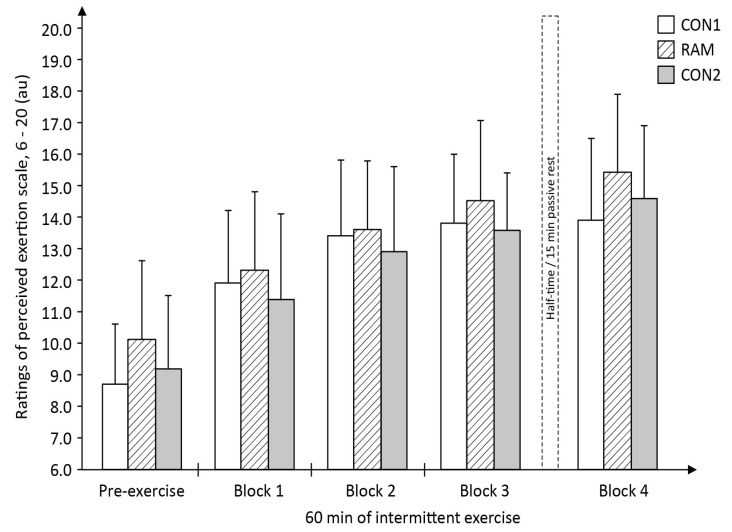
Players’ rating of perceived exertion (RPE) during the modified Loughborough Intermittent Shuttle Test (mLIST) exercise in the in the non-fasted or control (CON1 = before Ramadan month, CON2 = after Ramadan month) and Ramadan-fasted (RAM = in the Ramadan month) exercise trials (*N* = 14).

The RPE data indicate a main effect for time (*F*_4,52_ = 36.41, *P* < 0.001; Figure 8). Although RPE values during RAM trial were generally higher than those in CON1 and CON2 trials, the two-way ANOVA indicated no statistical significance for trial (*F*_2,26_ = 1.79, *P* = 0.19) and interaction (*F*_8,104_ = 1.03, *P* = 0.42). For the session-RPE, one-way ANOVA initially indicated statistical significance difference between trials (*F*_2,26_ = 4.01, *P* < 0.026). However, post-hoc test indicated no statistical significant difference among the three trials, albeit the RAM trial showed a slightly higher value (CON1: 4.1 ± 1.4 vs. RAM: 5.6 ± 1.6 vs. CON2: 4.7 ± 1.2 au; all *P* > 0.017).

### 4.6. Blood Glucose and Blood Lactate Concentrations

There were statistical significant primary effects of blood glucose concentration for trial (*F*_2,26_ = 4.92, *P* = 0.015; Table 3). Post-hoc analysis indicated that blood glucose was significantly different between RAM and CON2 trials at end of block 1 and block 2 (both *P* < 0.017) only. There were no significant differences between RAM and both CON trials at all other time-points (all *P* > 0.017). There was also significant difference in blood glucose for time (*F*_4,52_ = 22.39, *P* < 0.001), where blood glucose was elevated from the start of exercise and peaked after 30 min of exercise, and then slightly declined at the end of the 60-min exercise in all three experimental conditions. Blood glucose concentration also showed significant interactions effects (*F*_8,104_ = 5.43, *P* < 0.001). For blood lactate concentration, there were primary effects of trial (*F*_2,26_ = 4.12, *P* = 0.028) and time (*F*_4,52_ = 26.07, *P* < 0.001), but no interactions (*F*_8,104_ = 1.03, *P* = 0.42; Table 3). Blood lactate levels showed a similar pattern to that of blood glucose where the levels of blood lactate sharply increased from the start of exercise and levelled-off at the end of the first three blocks of exercise before decreasing slightly at end of block 4. The post-hoc analysis however, indicated no significant difference in blood lactate between RAM with either CON1 or CON2 trials across the 60-min exercise (all *P* > 0.017). Overall and most importantly, blood glucose and blood lactate concentrations were all within the expected or normal values commonly observed during exercise.

### 4.7. Body Mass, Fluid Intake and Sweat Volume

Body mass at pre- and post-exercise was not significantly different between the three experimental trials (all *F* ratios were *P* > 0.05; Table 4). Pre and post-exercise Usg data similarly, indicated no significance differences among the three exercise trials (all *F* ratios were *P* > 0.05). No fluid was consumed during exercise in the RAM trial. Fluid intake during both of the CON trials were shown to be equivalent (CON1: 930 ± 309 vs. CON2: 817 ± 320 mL; *P* = 0.16). One way ANOVA showed that percentage of sweat lost was not significantly different between the three experimental trials (CON1: 2.3 ± 0.7 vs. RAM: 2.5 ± 0.6 vs. CON2: 3.1 ± 1.3%; *F*_2,39_ = 3.02, *P* = 0.06).

### 4.8. Sleep Quantity, Daytime Sleepiness, Mood State and Perceived Readiness

In the Ramadan period, many of the players extended their overnight sleep till late morning on the day of their experimental trial. As a result, there were no significant differences between the three trials for the players’ sleep duration (*F*_2,39_ = 0.15, *P* = 0.87; Table 5). There was also lack of significant difference in the players’ daytime sleepiness as determined via the Karolinska Sleepiness Scale (*F*_2,39_ = 1.45, *P* = 0.25). There were no significant differences between the three experimental trials for any of six mood subscales in the BRUMS (all *F* ratios were *P* > 0.05; data not shown). Table 6 shows the players’ subjective responses to the pre-exercise questionnaires. There were no significant differences between the three trials in perceived readiness to compete, level of alertness and concentration (all *P* > 0.05). However, players in the fasted state (i.e., RAM trial) reported a significantly higher level of perceived tiredness as compared to the CON1 and CON2 trial (*F*_2,39_ = 4.28, *P* = 0.02). Post-hoc analysis indicated there were significant differences between CON1 and RAM (*P* = 0.016) but not between the CON2 and RAM trial (*P* = 0.52).

### 4.9. Training Load

Before Ramadan, the team trained 3–5 times per week and competed on Saturdays. For the period during Ramadan and two weeks after the Ramadan month, training was maintained but no competitive match was played. Competitive league matches were resumed three weeks after the end of the Ramadan month. Training programme throughout the study’s investigation period was planned and organized by the coaching staff with primary objective of maintaining the players’ fitness levels. One-way ANOVA indicated significant differences in training load within the three trials (*F*_2,39_ = 4.59, *P* < 0.016; Table 7). Post-hoc analysis, however, indicated there were only significant differences in training load between CON1 and CON2 trials (*P* = 0.016, *d* = 1.09, large). There were no significant differences in training load between CON1 and RAM (*P* = 0.13, *d* = 0.90, moderate) and between CON2 and RAM trials (*P* = 1.0, *d* = 0.31, small).

## 5. Discussion

The present study endeavoured to closely control for the confounding factors that plagued previous Ramadan and exercise performance studies such as differences in the participants’ pre-exercise food and fluid intake, sleep pattern and training stimulus between Ramadan-fasted and non-fasted exercise trials. In the present study, all these factors were equivalent between the CON and RAM trials. Despite the similarities, the study’s major finding was that players’ sprint performance was shown to be poorer (i.e., slower mean sprint times) throughout the 60-min intermittent exercise during RAM trial as compared to their sprint performance in CON1 and CON2 trials. Further, it was shown that the poorer sprint performance in the Ramadan-fasted state was not associated with any substantial differences in the players’ metabolic measures such as blood glucose or blood lactate concentrations or physiological effort (as assessed via mean HR during exercise) between CON and RAM trials throughout the mLIST. In addition, the MVIC tests results of both the exercised and non-exercised muscles were equivalent between pre-exercise and during the mLIST in the RAM trial; which indicate there was minimal degree of fatigue within the working muscles (i.e., peripherally-related) and in the neural activation of the muscles (i.e., centrally-driven pathways) that can help to explain for the poorer sprint performance throughout the 60-min intermittent exercise in the RAM trial. Overall, these findings together with the observation of an early onset of the sprints performance decrements within the first 15-min of exercise, provided good evidence of the substantiated view that the negative impact of Ramadan fasting on exercise performance was largely due to negative placebo (or nocebo) effects of observing the religious fast.

Previous studies have indicated a lowering in the players’ submaximal intensity component to conserve their efforts for the more critical high-intensity efforts when athletes are exposed to heat or hypoxia [29,30]. In the present study, all submaximal locomotor activities of walking, jogging, and striding were dictated by pre-planned beeping sounds and players had “control” only over their maximal sprinting efforts. Thus by design, players in the present study were not able to pace their submaximal running speeds. Sprinting constitutes only ∼1% to ∼12% of the distance covered during a football match [31] but this ability cannot be undermined since linear sprints are the most frequent action immediately preceding goal scoring opportunities in top-level matches [18]. Therefore, the capacity to perform sprints at the player’s top velocity over an entire match is a critical reflection of a player’s football performance. Thus, in this regard, the present study’s results clearly indicate that Ramadan fasting has a negative impact on football performance, and possibly performance in other intermittent team-sports as well.

The present study’s findings clearly supported previous findings in which high-intensity running distance covered were significantly lower during a 90-min football match played in the Ramadan state as compared to a match played in the non-Ramadan state [32]. In that study, performance decrement was similarly observed during the first 15-min of the 90 min match. This early “deterioration” in performance is unlikely to be due to the onset of physiologically-related fatigue as a result of factors such as total depletion of endogenous substrate, severe dehydration or metabolic disturbances within the muscles, but rather most probably be due to either a conscious or unconscious pacing strategy adopted by the players when in the fasted state. Perhaps, in the Ramadan-fasted state, players deliberately or were instinctively “compelled” via the Governor system [33] to moderate their sprinting efforts (which is the most physically demanding activity of the mLIST) throughout the exercise in order to conserve their available resources to ensure that they would be able to successfully complete the other submaximal shuttle runs.

A previous study showed that Ramadan fasting led to a decline in 5000 m running performance in well-trained runners [34]. The runners’ maximal strength via an isometric knee extension test performed in the Ramadan-fasted state was also negatively affected. The study’s investigators subsequently concluded that the decrease in leg strength could have led to the observed slower run times [34]. However, a limitation in that study was that both the running performance and isometric strength tests were conducted as independent trials, i.e., on separate occasions, and thus it cannot be ascertained with confidence that the poorer endurance performance in Ramadan was directly due to the declined in isometric leg strength. In contrast, the present study ensured that players’ maximal muscle tests were conducted within the 60-min intermittent exercise. If impaired muscle functions (e.g., strength) and/or related adverse metabolic response within the contracting muscles were the key mechanism(s) to the poorer exercise performance in the Ramadan-fasted state, a greater decreased in the MVIC of the knee would also be expected in the RAM trial as compared to that in the CON trials. Rather surprising, the MVIC of the knee extensor indicated that muscle functional capability (i.e., the ability to generate high levels of contraction) was unaffected by Ramadan fast (Figure 5) and is thus not a factor that could have led to the poorer sprinting performance in the RAM compared to that in the CON1 and CON2 trials.

Recently, Bouhlel and colleagues [35] showed that repeated 6 s cycle sprints times were slower in the Ramadan-fasted relative to non-fasted state; but this decrement in performance was not associated with any changes in the muscles’ electromyographic measures taken immediately after the last sprint effort. Subsequently, the study’s investigators argued that the reduction in their sprint performance must originate from fatigue occurring within the central nervous system (CNS) rather than within the musculature [35]. Similarly, in the present study, the decline in sprint performance in the RAM trial could be that Ramadan fasting magnifies the perception of fatigue during the mLIST resulting in a decrease in central drive relative to that perceived in the non-fasted state and this subsequently, reduces the CNS ability to maximally recruit all the muscle fibres during the all-out sprint efforts. This interpretation is partially supported by the higher pre-exercise subjective ratings of tiredness (Table 6) and overall higher levels of RPE throughout exercise (Figure 8) in RAM trial. However, the lack of change in the objective measures of both the MVIC of the leg (exercised muscles) as well as the MVIC of the hand-grip (non-exercised muscles) across the entire 60 min duration within the RAM trial (Figure 5 and Figure 6, respectively) did not seem to support this theory of a higher level of CNS fatigue in the Ramadan-fasted state. If central-pathways fatigue was the primary cause of the poorer performance in sprints in RAM, there should be a decrease in the MVIC of the hand-grip as well, i.e., a ‘fatigued’ CNS should also lead to a decline in voluntary contraction of the non-exercised (i.e., the less fatiguing arm) muscle. This was not the case in the present study. Therefore, by the process of elimination, it can be deduced that the primary cause of the poorer performance in the sprints during RAM trial is not due to either peripheral- or CNS-mediated fatigue, but rather most likely to be due to the negative effects of placebo. This view has credence as neural activation from the CNS as the primary driver to single maximal or explosive effort during exercise, and coupled with the fact that the poorer sprint performance in RAM trials was observed as early as within the first 15 min (i.e., in exercise block 1) of the mLIST. In short, fasted individuals moderated their sprinting efforts early during the mLIST exercise, either consciously or subconsciously, as a way of coping with the challenging demands of completing the 60-min intermittent exercise in the Ramadan-fasted state without catastrophic consequences later in the mLIST [36]. It may therefore be deduced that the decline in sprint performance during the RAM trial was not due to any physiological-induced mechanism from the act of Ramadan fast but rather due to the players pacing their efforts as a result of placebo effects, or in the present study’s situation, aptly termed nocebo consequences of observing the Ramadan fast [37,38].

Nonetheless, during prolonged exercise, it may be that the “fatigue” experienced by an athlete is perhaps, physiologically different during the early stage of exercise compared to the late stage of the exercise [39,40,41]. In the present study, late in the RAM exercise trial, it might be that muscle glycogen concentration was reduced to critical levels leading to the poorer sprint performance. Krustrup and colleagues [40] showed that despite ingesting breakfast, as well as a meal two hours prior to a football match, muscle glycogen decreased significantly, with about ~50% of muscle fibres being completely or almost depleted of muscle glycogen after the match, Further, they showed that reduced muscle glycogen stores were associated with a decline in physical exertions in the second half of the match [40]. It should also be noted that the players in the present study was exercising in a hot, humid environment, which could have led to a greater rate of usage of endogenous muscle glycogen [42] leading to its rapid depletion within specific muscle fibres, i.e., fast-twitch fibres [43]. While this muscle glycogen limiting argument is reasonable, this might again, not be the case in the present study for several reasons. Firstly, it unlikely that the players in the Ramadan-fasted state were possessing lower levels endogenous muscle glycogen at pre-exercise relative to that in the CON trial. Although endogenous muscle glycogen was not directly measured, the players were optimally provided with sufficient carbohydrates (and total calories) in the 24 h period prior to the RAM trial and previous studies have indicated minimal impact of fasting on endogenous muscle glycogen levels in as long as no strenuous physical activity is engaged during the duration of the fast. Indeed, endogenous muscle glycogen depleted by a mere 25% as a result of fasting for a 24-h period [44,45]. Moreover, firstly the present study’s players fasted for only ~12 h and they did not engage in any strenuous physical activity prior to the exercise trial during the fasted period. Secondly, the mLIST exercise was only of 60 min rather than 90 min duration. A review on the effect of duration of exercise on muscle glycogen usage suggest that muscle glycogen tended to lower to critical levels only after >60 min of moderate to high-intensity exercise [46]. Thirdly, blood lactate concentration, which has been considered as an indirect indicator of the substrate breakdown due to glycolysis [47], was not significantly different between CON and RAM trials at the start and at the end of each four exercise blocks (Table 3). All of these indicate that the muscle glycogen levels in the Ramadan-fasted state was not compromised throughout the mLIST exercise.

However, particularly towards the late part of the 60-min mLIST, dehydration may however adversely impact muscle function (i.e., on sprints performance) via a diminished ability of the CNS to recruit motor units efficiently. In particular, the loss of large amount of electrolytes through sweating can cause ionic imbalances [48] resulting in the disruption of the neuromuscular function of the working muscles. Edwards and Noakes [39] also suggested that dehydration may result in an unconscious effort to reduce subsequent physical exertions to try to maintain homeostasis and protect against intracellular disturbances. And when dehydration is accompanied with hyperthermia, as the case might be towards the end of the 60 min mLIST exercise, it can exert a more profound effect on muscular functions and this could have resulted in the greater slowed sprinting speeds in the RAM trial. However, the last 15 min of exercise (i.e., block 4) showed that sprinting times has reverted close to sprint times observed in block 1—further indicating that there was minimal accumulation of extreme physical fatigue in the Ramadan-fasted state.

A high body core temperature, instead of substrate availability, has been identified as a possible limiting factor to exercise performance in the heat [49]. For example, the increased heat stress and high core temperatures from exercise have been reported to reduce the player’s ability to perform repeated high-intensity sprints [50]. Players in the RAM trial did not consume fluid for at least ~12 h before exercise (resulting in hypohydration) and were not allowed to consume fluids during exercise (leading to dehydration), and hence it was expected that the thermoregulatory functions and/or processes of the players in the RAM trial would be compromised relative to the CON trials, which in turn could adversely influenced their subsequent exercise performance, vis-a-vis causing the fasted players to slow down in their sprinting speeds [36]. Rather surprisingly, there were no significant differences in both mean *T*_re_ and *T*_sk_ in both the CON trials to that in the RAM trial (Table 2). The *T*_re_ data in all trials showed that none of the values surpassed the 40 °C mark, which is deemed to be the critical threshold point for internal core temperature [49]. It should note however that the present *T*_re_ are not peak values since *T*_re_ was recorded only at the end of each 15 min exercise block and moreover in RAM trials, fasted players performed less intense work. It remains possible that there were instances during the mLIST where *T*_re_ may have breached the 40 °C mark, which could have led to some altercations in the players’ exercise performance at that time point. Prolonged exercise in hot environmental conditions can lead to a rise in internal core temperature that can result not only in the decline in exercise performance, but in the worst case where the exercising individual succumbing to heat illness [51]. Nonetheless, the present core and skin temperature data do suggest that fasted players can physiologically adjust their exertions and exercise safely in a hot humid environment. The present study to our knowledge, is the first to monitor the *T*_re_ and *T*_sk_ of Ramadan-fasted individuals throughout a prolonged exercise (>60 min) in the hot and humid environment, and given the present study’s mixed findings, further research in this area is warranted.

Yet another possible contributor to the reduction in performance is the elevation of negative mood states and suppression of positive feelings [52]. These negative feelings can permeate via poor sleep quality and/or quantity and/or drastic shift in the body’s circadian rhythm during Ramadan [9,10]. Indeed, sleep loss during Ramadan has been reported to be closely associated with detrimental effects on mood and perception [8,9]. The present data however showed that sleep hours for the evening before the RAM experimental day, assessed objectively, was however equivalent to that in the two CON trials (Table 5). During Ramadan, the players consumed their *sahur* meal at ~05:30 and then went back to bed thereafter to continue with their sleep until late morning as was similarly observed in a group of professional football players [53]. Detraining, as a result of reduced training load or stimulus due to a lowering of training frequency or intensity during the Ramadan month has also been highlighted as another possible cause of lowered performance in fasted Muslim athletes [11,12]. In the present study however, training load for the week before all three experimental trials, although was slightly lowered in RAM and CON2 trials when compared to CON1, was generally equivalent to each other (Table 7).

In summary, the slower sprint times in RAM relative to CON trials were not due to physical fatigue from energy deficiency since blood glucose and blood lactate concentrations values throughout the exercise were generally similar between RAM and CON trials, or loss in muscular generating capabilities since no difference in the players’ MVIC of the knee extensor (exercised muscle) was observed between CON and RAM trials. The greater sprints decrement in RAM was also not likely to be due to greater fatigue within the CNS pathways since there was no drastic change in the hand-grip (non-exercised muscle) MVIC measure throughout the RAM trial. Given these observations and the fact that the decrement in sprints performance was already evident very early in the mLIST (which precludes the involvement of the peripheral limitations such as lack of endogenous substrate or excessive dehydration), it is thus reasoned that the negative effect of RAM on physical performance is primarily due to placebo (or nocebo effects) of performing the Ramadan fast per se. Nocebo is defined as the change arising from an individual’s negative perceptions about the likely outcome from receiving a (negative) treatment, vis-à-vis, observance of the Ramadan fast [37,38]. Players in the present study have been observing Ramadan fast annually for many years. The individual’s prior experience as to how he feels when exercising in the Ramadan-fasted state (presumably negative experience since exercise in the Ramadan-fasted state is physiologically challenging [1,2]) would have played a contributory role or even reinforced the nocebo response. Thus the players’ own expectations of a poorer performance is deemed as the primary cause of the relatively slower sprints in the RAM trial. Owing to the difficulty in defining the boundaries of consciousness, it is uncertain whether the fasted players were deliberately or unconsciously lowering their sprint efforts during the mLIST, in particular during the early stages of the mLIST. Interestingly, it was calculated that the percentage differences in the mean sprint times between RAM and both CON trials ranged between 2.9% to 5.5% across the four exercise blocks (Figure 4), which were within the magnitude of the impact placebo, estimated to be 1% to 5% [37].

## 6. Conclusions

Control for confounding factors such as the participants’ pre-exercise dietary intake, sleep quality and training load were strong features of this investigation; and these factors were shown to be equivalent in the Ramadan-fasted and non-fasted conditions. Despite this equivalence, the results still showed that Muslim football players’ sprinting performance declined to a greater extent throughout the 60-min intermittent exercise in the Ramadan-fasted state as compared to their sprints performance in the non-fasted state.

## 7. Practical Implications

If competing in the Ramadan month is unavoidable, specific strategies such as pre-match cooling (e.g., cold-water immersion, body cooling vest, etc.) and frequent mouth-rinsing during match, are recommended to attenuate the influence of Ramadan fasting on physical and cognitive performances [54,55]. It is also important that the coach educates and raises the awareness of the nocebo responses during exercise in the Ramadan-fasted state and organize friendly matches prior to competition where players can rehearse playing in the fasted state. This will help the fasted player learn to cope and adapt with the physiological effects of exercise in a challenging condition and conscientiously manage the distribution of his efforts throughout a match in Ramadan.

## Figures and Tables

**Table 1:** Meal distribution patterns of the pre-packed food and fluids over the 24 h period prior to each exercise trial in the non-fasted or control (CON1 = before Ramadan month, CON2 = after Ramadan month) and Ramadan-fasted (RAM = in the Ramadan month) periods in the study.

CON1 and CON2 Trials	RAM Trial
Day prior to test day	Day prior to test day
Meal 1 consumed at ~19:45 (i.e., dinner)	Meal 1 consumed at ~19:45 (i.e., *iftar*)
	Meal 2 consumed at ~23:00 (supper)
Test day	Test day
Meal 2 consumed at ~07:00 (breakfast)	Meal 3 consumed at ~05:30 (*sahur*)
Meal 3 consumed at ~13:00 (lunch)	

*sahur* = meal sitting at the start of day’s fast; *iftar* = meal sitting at the end of the day’s fast. Note: Subjects were instructed to consume their three meals at the above specify times; however actual meal-sitting times varied (range from ~20 to 30 min during the CON and from ~15–20 min during the RAM periods).

**Table 2:** Players’ rectal and skin temperature measured at the start and at the end of each exercise block during the modified Loughborough Intermittent Shuttle Test (mLIST) exercise in the non-fasted or control (CON1 = before Ramadan month, CON2 = after Ramadan month) and Ramadan-fasted (RAM = in the Ramadan month) trials (*N* = 11).

	Pre-exercise	Block 1	Block 2	Block 3	Block 4
Rectal (°C):					
CON1	37.75 ± 0.30	39.16 ± 0.46	39.67 ± 0.30	39.82 ± 0.46	39.49 ± 0.39
RAM	37.94 ± 0.24	39.07 ± 0.43	39.58 ± 0.38	39.80 ± 0.35	39.71 ± 0.32
CON2	37.98 ± 0.37	38.97 ± 0.32	39.54 ± 0.51	39.56 ± 0.55	39.43 ± 0.41
Skin (°C):					
CON1	34.22 ± 1.32	34.66 ± 0.99	34.34 ± 1.02	34.07 ± 0.73	33.62 ± 1.87
RAM	34.66 ± 0.67	34.61 ± 0.91	34.59 ± 1.06	34.41 ± 1.15	33.80 ± 1.58
CON2	33.56 ± 0.84	34.08 ± 0.58	34.17 ± 0.84	33.83 ± 0.77	33.61 ± 0.81

**Table 3:** Players’ blood glucose and blood lactate concentration at the pre-exercise and at the end of each block of exercise during the modified Loughborough Intermittent Shuttle Test (mLIST) exercise in the non-fasted or control (CON1 = before Ramadan month, CON2 = after Ramadan month) and Ramadan-fasted (RAM = in the Ramadan month) trials (*N* = 14).

	Pre-Exercise	Block 1	Block 2	Block 3	Block 4
Blood glucose concentration (m·mol^−1^):					
CON1	6.8 ± 1.1	7.0 ± 1.3	7.6 ± 0.8	7.2 ± 1.0	6.2 ± 1.0
RAM	6.2 ± 0.5	7.4 ± 0.7 ^†^	8.3 ± 1.1 ^†^	7.8 ± 1.1	6.0 ± 0.5
CON2	6.7 ± 1.2	6.3 ± 1.3	7.0 ± 0.9	6.8 ± 0.8	5.7 ± 0.7
Blood lactate concentration (m·mol^−1^):					
CON1	3.3 ± 0.6	7.9 ± 2.3	7.6 ± 2.6	6.9 ± 2.9	6.2 ± 2.6
RAM	2.8 ± 0.6	6.9 ± 2.3	6.9 ± 3.0	6.8 ± 2.4	6.4 ± 1.4
CON2	2.4 ± 1.2	6.2 ± 2.8	6.5 ± 2.8	6.4 ± 3.4	5.6 ± 2.9

^†^ Post-hoc significant difference between RAM and CON2 trial, *P* < 0.017.

**Table 4:** Players’ pre- and post-modified Loughborough Intermittent Shuttle Test (mLIST) exercise body mass and hydration status in the non-fasted or control (CON1 = before Ramadan month, CON2 = after Ramadan month) and Ramadan-fasted (RAM = in the Ramadan month) trials (*N* = 14).

	Pre-Exercise	Post-Exercise
Body mass (kg):		
CON1	67.1 ± 11.3	66.5 ± 11.2
RAM	65.7 ± 11.1	64.1 ± 10.7
CON2	66.4 ± 11.3	65.1 ± 11.1
Urine specific gravity (au):		
CON1	1.021 ± 0.002	1.021 ± 0.006
RAM	1.021 ± 0.007	1.023 ± 0.005
CON2	1.021 ± 0.006	1.019 ± 0.006

**Table 5:** Sleep duration and level of daytime sleepiness of players in the non-fasted or control (CON1 = before Ramadan month, CON2 = after Ramadan month) and Ramadan-fasted (RAM = in the Ramadan month) trials (*N* = 14).

	Duration of Sleep the Night Prior to Exercise Trial (h)	Karolinska Sleepiness Scale Score (au)
CON1	7.5 ± 1.0	3.3 ± 0.9
RAM	7.9 ± 2.8	4.2 ± 1.6
CON2	7.7 ± 1.9	3.8 ± 1.7

au = arbitrary unit.

**Table 6:** Players’ subjective responses to the pre-exercise questionnaires in the non-fasted or control (CON1 = before Ramadan month, CON2 = after Ramadan month) and Ramadan-fasted (RAM = in the Ramadan month) trials (*N* = 14).

	Perceived Level of Readiness to Exercise (mm)	Perceived Level of Tiredness (mm)	Perceived Level of Alertness (mm)	Perceived Level of Concentration (mm)
CON1	30.8 ± 25.0	20.4 ± 14.5	79.6 ± 17.7	83.6 ± 13.2
RAM	35.2 ± 22.6	44.2 ± 25.1 ^†^	70.6 ± 23.1	76.5 ± 17.2
CON2	42.3 ± 22.0	35.1 ± 24.2	70.4 ± 20.8	76.4 ± 15.3

^†^ Post-hoc significant difference between CON1 and RAM trial, *P* < 0.017.

**Table 7:** Training variables for the week before the non-fasted or control (CON1 = before Ramadan month, CON2 = after Ramadan month) and Ramadan-fasted (RAM = in the Ramadan month) exercise trials (*N* = 14).

	CON1 Trial	RAM Trial	CON2 Trial
Number of sessions over the week	4 ± 1	4 ± 1	3 ± 1
Training load over the week (au)	2136 ± 233 ^†^	1914 ± 258	1821 ± 345

au = arbitrary unit; ^†^ Post-hoc significant difference between CON1 and CON2 trials, *P* < 0.017; Training load over the week is the sum of the all training sessions performed for the entire week.
